# Pneumococcal conjugate vaccination in The Gambia: health impact, cost effectiveness and budget implications

**DOI:** 10.1136/bmjgh-2021-007211

**Published:** 2021-12-16

**Authors:** Clint Pecenka, Effua Usuf, Ilias Hossain, Sana Sambou, Elisabeth Vodicka, Deborah Atherly, Grant Mackenzie

**Affiliations:** 1 Center for Vaccine Innovation and Access, PATH, Seattle, Washington, USA; 2 Disease Control and Elimination, Medical Research Council The Gambia, Banjul, Gambia; 3 Ministry of Health, Government of the Gambia, Banjul, Gambia; 4 Disease Control and Elimination, MRC Unit The Gambia at LSHTM, Banjul, Gambia; 5 London School of Hygiene & Tropical Medicine, London, UK

**Keywords:** immunisation, health economics, pneumococcal disease

## Abstract

**Introduction:**

Introducing pneumococcal conjugate vaccine (PCV) in many low-income countries has contributed to reductions in global childhood deaths caused by *Streptococcus pneumoniae*. Many low-income countries, however, will soon reach an economic status leading to transition from Gavi, the Vaccine Alliance vaccine funding support and then face increased expenditure to continue PCV programmes. Evaluating the cost-effectiveness of PCV in low-income countries will inform such country decisions.

**Methods:**

We used empiric data on the costs of vaccine delivery and pneumococcal disease and PCV programme impact on disease among children less than 5 years old in The Gambia. We used the UNIVAC cost-effectiveness modelling tool to compare the impact and cost-effectiveness of pneumococcal conjugate vaccination to no vaccination over 20 birth cohorts starting in 2011. We calculated costs per disability-adjusted-life-year (DALY) averted from government and societal perspectives and undertook scenario and probabilistic sensitivity analysis.

**Results:**

We projected that, over 20 years, PCV in The Gambia could avert 117 000 total disease episodes in children less than 5 years old, including outpatient and hospitalised pneumonia, pneumococcal sepsis and meningitis (including sequelae). Vaccination could avert 9000 outpatient pneumonia visits, 88 000 hospitalisations and 3300 deaths due to pneumonia, meningitis and sepsis. Approximately 100 000 DALYs are expected to be averted. Averted visits and hospitalisations represent US$4 million in healthcare costs expected to be saved by the government and US$7.3 million if household costs are included. The cost of the vaccination programme is estimated at US$2 million. In the base scenario, most alternative scenarios and nearly 90% of the probabilistic scenarios, pneumococcal vaccination is cost saving in The Gambia.

**Conclusion:**

Pneumococcal conjugate vaccination is expected to generate substantial health gains and is likely to be cost saving in The Gambia. Policymakers in similar settings should be confident to maintain their PCV programmes.

**Key questions**

**What is already known?**

- Pneumococcal conjugate vaccines (PCV) are effective and introduction in many low-income countries has contributed to substantial reductions in pneumococcal deaths.
- Approximately 45% of the global birth cohort are unable to access PCV primarily due to issues of cost.
- Most studies, including those in low-income countries, have found PCV to be highly cost-effective in the local context but not necessarily cost-saving.
- Many low-income countries are approaching the economic threshold at which they will begin to transition from Gavi support, with a required increase of country expenditure to continue PCV programmes.

**What are the new findings?**

- The Gambian PCV programme is likely to be cost-saving between 2011 and 2030, with projected reductions in disease burden (88 000 hospitalisations and 3300 deaths) and savings of US$4 million for medical care.
- We estimated that the PCV programme will remain cost-saving until the country pays approximately US$0.66 per dose.
- Even after The Gambia incurs the full cost of vaccine purchase (US$2.95 per dose), the PCV programme is likely to remain highly cost effective.

**What do the new findings imply?**

- This is the first study to indicate that PCV immunisation, at actual levels of payment for vaccine and provision of healthcare, can be cost-saving at the country level.
- Low-income countries should be confident in investing in PCV despite financing challenges because overall government spending is likely to be reduced by vaccination.

## Introduction

An estimated 318 000 global pneumococcal deaths occurred in children in 2015.^1^ In Africa between 2000 and 2015, pneumococcal deaths were estimated to have fallen by 63% from 447 000 to 166 000. In The Gambia, a vaccine probe trial estimated that at least 16% of all childhood deaths were caused by *Streptococcus pneumoniae*.^2^ Beyond health implications, treating pneumonia and other pneumococcal diseases can be costly to families and health facilities. In The Gambia, each outpatient visit costs society more than US$10 with hospitalisation costs typically exceeding US$100.^3^ These are substantial costs in a country with a Gross National Income (GNI) per capita of US$750 per year.^4^

The introduction of pneumococcal conjugate vaccines (PCV) in many low-income countries is one of the major reasons for the global progress against pneumococcal disease. The Gambia introduced seven-valent PCV (PCV7) in 2009, which was later replaced by PCV13 in 2011. Population-based surveillance from 2008 to 2017 in the Basse Health and Demographic Surveillance System (Basse HDSS) in rural Gambia compared disease incidence before and after PCV introduction.^5–7^ The incidence of invasive pneumococcal disease in children younger than 5 years of age declined by 80%, from 184 to 38 cases per 100 000 person-years and pneumonia hospitalisation declined by 27%, from 43 to 32 cases per 1000 person-years.^8^ Empiric data on the impact of PCV programmes in Africa are also available from Kenya as well as other countries around the world.^9 10^ To date, 60 low-income and middle-income countries have introduced PCV into their national immunisation programmes with support from Gavi, the Vaccine Alliance (Gavi).^11^ Many cost-effectiveness studies have demonstrated the value of PCV vaccination.^12–14^ Only a few of these studies, however, address cost-effectiveness in low-income countries.^15–18^ These studies in Kenya, Uganda, Ethiopia and The Gambia have projected that PCV will be a cost-effective health investment, even under very different pricing assumptions and willingness to pay thresholds. Local costing data for these studies, however, were limited and vaccine impact was based on assumptions and modelling rather than observed data. A study from Kenya based on the observed impact of PCV and including local costing data also concluded that continuing PCV was cost-effective, even after transition from Gavi support.^19^

Many African countries have introduced PCV with the support of Gavi and its pneumococcal Advance Market Commitment (AMC), which reduces the price of PCVs. Gavi’s cofinancing policy stipulates that initial self-financing countries pay US$0.20 per dose of PCV while Gavi pays the balance of the full price, which is currently between US$2.00 and US$3.30 depending on the product.^20 21^ Over time, as income per capita increases, countries transition from an initial self-financing phase to preparatory transition, accelerated transition, and, finally, the fully self-financing phase. At each stage, countries pay a larger share of the full AMC price and, on reaching the fully self-financing phase, pay 100% of the AMC price. Each country that has introduced PCV vaccination faces decisions on whether and how to increase spending on PCV to maintain their programmes as they transition from Gavi support. Economic considerations may have influenced decisions by India, Sri Lanka, Timor-Leste and Vietnam not to introduce PCV, even though they are eligible to self-finance the AMC price.^22^

Given the importance of economic considerations in PCV policy decisions in low-income countries, local cost-effectiveness data are needed. We aimed to evaluate the cost-effectiveness and budget implications of PCV in The Gambia using locally observed programme impact, cost of illness, and vaccine programme cost data.

## Methods

### Modelling approach

The estimated health impact of PCV was based on the observed vaccine impact in The Gambia.^8^ The UNIVAC model framework (V.1.3.7) was then used to estimate the impact and cost-effectiveness of PCV at the national level under different vaccine pricing scenarios, and different assumptions about the underlying burden of disease. Model inputs included disease burden, healthcare utilisation and costs, as well as vaccine cost, coverage and the observed impact of PCV. UNIVAC is a proportionate outcomes model that, along with its predecessor (TRIVAC), has been widely used. The most recent model version is available online.^23^

This analysis examines the impact of PCV introduction in The Gambia compared with an alternative of no pneumococcal vaccination from 2011 to 2030. We make this comparison to demonstrate the current vaccination programme’s health impact and cost effectiveness to inform local and regional policymakers of the value of this investment. We modelled vaccination in 20 consecutive birth cohorts and project health and cost outcomes for each cohort over a 5-year period, though some longer-term sequelae costs were included for 10 years. Modelling vaccination over 20 birth cohorts allowed us to examine the benefits over the foreseeable life of the programme and its anticipated costs to The Gambia. The Gambia’s GNI per capita is US$750 in 2019 and faces global economic headwinds due to a pandemic.^4^ The Gavi transition policy currently requires the average GNI per capita to exceed US$1036 over 3 years before The Gambia will dramatically increase its contributions to vaccine purchases.^4 20^ We include a scenario, however, to examine this possibility. Though treatment costs for young children in The Gambia are fully funded by the government, this study evaluates costs from both the government and societal perspectives. We examine government and household costs so we have a more comprehensive picture of vaccination programme impact, including household expenses that occur prior to facility attendance, at the facility, and after discharge.

In addition to the cost of the vaccination programme and the cost of care averted, primary outcomes of interest were cases, outpatient visits, inpatient hospitalisations, deaths and disability-adjusted life years (DALYs) averted by the PCV programme. DALYs are utilised in this analysis given their preponderance in economic evaluations in low-income and middle-income countries. We examined these outcomes and the costs for non-severe outpatient pneumonia (all-cause), severe inpatient pneumonia (all-cause), pneumococcal sepsis and pneumococcal meningitis. We assumed non-severe pneumonia resulted in outpatient visits followed by recovery while severe pneumonia resulted in inpatient hospitalisation followed by recovery or death. Pneumococcal sepsis resulted in inpatient hospitalisation followed by recovery or death while pneumococcal meningitis resulted in inpatient hospitalisation followed by recovery with or without sequelae or death. Meningitis sequelae were modelled as neurological sequelae that impairs daily function, which is the most commonly observed condition following meningitis in The Gambia. All cases were included in modelled outcomes while costs were only applied to cases that sought care. A model schematic is available in the appendices.

Both costs and DALYs were discounted at 3% per year.^24^ Cost data were adjusted to 2017 US$ by reconverting back to Gambian Dalasis in the currency year in which data were collected, adjusting for inflation based on the Gambian consumer price index and then adjusting back to 2017 US$.^25^ Currency conversions were made using official exchange rates per the World Bank.^25^

### Burden of disease and health service utilisation

We modelled burden of disease and health services utilisation associated with non-severe pneumonia, severe pneumonia, pneumococcal sepsis and pneumococcal meningitis (including sequelae). For each category, we estimated numbers of cases, outpatient visits, inpatient hospitalisations and deaths. Deaths were not attributed to non-severe pneumonia or meningitis sequelae in our model. Disease incidence before and after the introduction of PCV was derived from passive population-based surveillance for pneumonia, sepsis and meningitis in the Basse HDSS from 2008 to 2017.^8^ Surveillance only captured those individuals that sought treatment and, thus, was an underestimate of total disease burden. Therefore, we calculated the total disease burden assuming that a percentage of non-severe pneumonia, severe pneumonia, pneumococcal sepsis and pneumococcal meningitis cases went unreported and did not access care. We estimated total disease burden by scaling disease incidence data by the inverse of the access to care rate for each clinical category. Access to care for each clinical category was calculated assuming constant disease incidence in the population but decreased access with greater distance to the nearest health facility.^26^ Disease incidence for each clinical category was calculated in five strata of distance from the nearest health facility. Access to care ratios for each distance stratum was calculated relative to the incidence in the stratum nearest to health facilities. Overall access to care for each clinical category was a population-weighted sum of the stratum level ratios.^27^ Access to care varied by disease category. We estimated that care was sought in 75% of cases of non-severe pneumonia, 82% of severe pneumonia cases, 94% of pneumococcal sepsis cases and 82% of pneumococcal meningitis cases. We applied the observed hospital case fatality rate for severe pneumonia (3.7%) to 82% of cases that reach the facility.^7^ We applied a conservative 5% case fatality rate to severe cases that did not reach the facility. We used local data estimating case fatality for hospitalised cases of meningitis (33%) and sepsis (8.1%).^8^ In the base case, we conservatively assumed the same case fatality rate for cases of meningitis or sepsis that did not seek treatment. Fifty-five percent of surviving meningitis cases resulted in neurological sequelae.^3^ The age distribution of disease for non-severe pneumonia, severe pneumonia, meningitis and sepsis was defined based on hospital surveillance data linked to child age.^8^ Burden of disease data were varied in alternative scenarios in accordance with CIs from the surveillance data. DALY weights for each disease outcome were taken from the 2016 Global Burden of Disease study and ranged from 0.051 for non-severe pneumonia to 0.402 for meningitis sequelae.^27^ The duration of illness was assumed to range from 6 days for non-severe pneumonia to 10 years for meningitis sequelae. Detailed burden of disease inputs are displayed in table 1.

**Table 1:** Input parameters for disease burden

Parameter	Estimate	Scenarios		Source/s
		Low	High	
Annual incidence per 100 000 age<5 years				
Non-severe pneumonia cases	2623.5	2361.3	2905.5	8 26
Non-severe pneumonia outpatient visits	1967.6	1705.0	2230.0	8
Severe pneumonia cases	4313.9	3992.1	4655.5	8 26
Severe pneumonia hospitalisations	3537.4	3106.0	3969.0	8
Pneumonia deaths	168.7	83.58	337.32	8
Pneumococcal meningitis cases	14.7	2.2	37.0	8 26
Pneumococcal meningitis hospitalisations	12.1	1.8	30.3	8
Pneumococcal meningitis deaths	4.6	0.7	11.4	8
Pneumococcal meningitis sequelae cases	8.1	1.2	20.3	3
Pneumococcal sepsis cases	154.8	110.5	211.0	8 26
Pneumococcal sepsis hospitalisations	145.5	103.9	198.4	8
Pneumococcal sepsis deaths	12.8	6.4	25.6	8
Disability weight for DALY calculations				
Non-severe pneumonia	5.1%	–	–	27
Severe pneumonia	13.3%	–	–	27
Pneumococcal meningitis	13.3%	–	–	27
Pneumococcal meningitis sequelae	40.2%	–	–	27
Sepsis	13.3%	–	–	27
Mean duration of illness (in days)				
Non-severe pneumonia	6	–	–	Assumption
Severe pneumonia	9	–	–	Assumption
Pneumococcal meningitis	13	–	–	Assumption
Pneumococcal meningitis sequelae	10 years	–	–	Assumption
Pneumococcal sepsis	10	–	–	Assumption
Age distribution of non-severe pneumonia cases				
<6 months	22%	–	–	8
<12 months	56%	–	–	8
<24 months	90%	–	–	8
<36 months	93%	–	–	8
<48 months	97%	–	–	8
<59 months	100%	–	–	8
Age distribution of severe pneumonia cases				
<6 months	24%	–	–	8
<12 months	60%	–	–	8
<24 months	90%	–	–	8
<36 months	93%	–	–	8
<48 m:	97%	–	–	8
<59 m:	100%	–	–	8
Age distribution of pneumococcal meningitis cases and sequelae				
<6 months	16%	–	–	8
<12 months	41%	–	–	8
<24 months	81%	–	–	8
<36 months	88%	–	–	8
<48 months	94%	–	–	8
<59 months	100%	–	–	8
Age distribution of pneumococcal sepsis cases				
<6 months	16%	–	–	8
<12 months	41%	–	–	8
<24 months	81%	–	–	8
<36 months	88%	–	–	8
<48 months	94%	–	–	8
<59 months	100%	–	–	8

DALY, disability-adjusted-life-year.

### Vaccine coverage

In The Gambia, PCV is scheduled at 2, 3 and 4 months of age. Coverage and vaccination timeliness data derive from a recent analysis evaluating The Gambia’s Expanded Programme on Immunisation and range from 97.8% for diphtheria/tetanus/pertussis vaccine (DTP1)/PCV1 to 92.3% for DTP3/PCV3.^28^ Coverage was assumed constant throughout the period of analysis.

### Programmatic impact on disease

The impact of PCV introduction was estimated from 10 years of population-based surveillance (from 2008 until 2017) in the Basse HDSS.^8^ Programme impact was calculated dividing the incidence of disease in the final 2 years by the incidence in the first 2 years of surveillance. Using the final 2 years of surveillance, data mitigated potential concerns about transitory impact on disease. These data are described in detail elsewhere.^8^ We calculated programme impact separately for non-severe pneumonia, severe pneumonia, pneumococcal sepsis and pneumococcal meningitis. The respective impact on disease was 5% (95% CI: 1 to 9), 27% (95% CI: 22 to 31), 95% (95% CI: 9 to 99) and 84% (95% CI: 75 to 89). Due to the design of the study, we used population level impact values over the first 5 years of life.^8^

### Vaccine programme costs

The Gambia cofinances PCV at US$0.20 per dose including an additional 5% increase in quantities due to wastage.^20 29^ In addition to vaccine costs, we estimated the incremental system cost per dose delivered to be US$0.33 based on data previously collected in The Gambia.^3^ These per dose system costs included both recurrent costs and capital costs. Recurrent costs included labour, syringes, safety boxes, cold storage operation and transport. Annual capital costs were obtained by annualising over a 20-year time horizon. Capital costs included cold storage, waste management, training, social mobilisation, stationery and monitoring. Per dose calculations were based on overall system costs and the number of fully immunised children.

### Health service costs

Treatment cost data were provided by Usuf *et al*.^3^ Children under 5 years of age participated in the study. Inpatient and outpatient treatment costs were reported for severe and non-severe pneumonia, pneumococcal sepsis and bacterial meningitis. Costs for non-severe pneumonia cases were considered as outpatient care and costs for severe pneumonia cases were considered as inpatient care. All sepsis and meningitis cases were assumed to require inpatient care. Costs associated with meningitis sequelae were calculated to occur annually for 10 years based on Usuf *et al*.^3^ While treatment is free to children in The Gambia, household costs can still be a burden and were included. Cost categories included staff, clinical consumables, drugs, intravenous fluids, oxygen therapy and hospital (hotel) costs. Costs to the household included prefacility pharmacy costs, traditional healers, transport, meals for family/visitors and lost time. We assumed that meningitis sequelae and disability costs of US$45 per year were borne by the household.^3^ Detailed cost data are reported in table 2.

**Table 2:** Input parameters for health service costs (2017 US$)

Parameter	Estimate	Scenarios		Source/s
		Low	High	
Health service costs				
Government cost per outpatient pneumonia visit	$6.82	$3.68	$7.45	3
Societal cost per outpatient pneumonia visit	$13.82	$5.74	$16.42	3
Government cost per pneumonia hospitalisation	$57.44	$34.29	$64.89	3
Societal cost per pneumonia hospitalisation	$97.48	$56.55	$116.42	3
Government cost per bacterial meningitis hospitalisation	$110.94	$67.05	$124.68	3
Societal cost per bacterial meningitis hospitalisation	$152.86	$105.83	$209.32	3
Government cost per pneumococcal sepsis hospitalisation	$77.73	$44.97	$106.64	3
Societal cost per pneumococcal sepsis hospitalisation	$128.99	$66.70	$173.78	3
Annual societal cost per bacterial meningitis sequelae	$44.88	$0	$44.88	3

### Alternative scenarios

The base case scenario included the best available data inputs, including local vaccine impact, cost of illness and cost of delivery estimates. To assess the robustness of our results, we conducted scenario analyses adjusting key input parameters. Parameters were selected based on their potential influence on the results or perceived uncertainty. The additional scenarios included upper and lower bounds of disease incidence for each disease category. Variation in disease incidence translated to increases or decreases in outpatient visits, hospitalisations and deaths. We then examined scenarios with lower and higher treatment seeking rates by reducing or increasing baseline rates for each disease category by 10 percentage points or up to a maximum of 100%. To further explore the impact of burden on our results, we halved and doubled baseline case fatality rates. We also examined a decrease in the impact of the PCV programme for each endpoint based on the CIs of the impact study.^8^ We examined a higher vaccine price of US$2.95 reflecting the price Gavi paid for the four-dose presentation of PCV used in The Gambia and separately modelled a scenario in which The Gambia transitions from initial self-financing to preparatory transition and begins incurring additional vaccine costs in 2025;^21^ a per dose systems cost of US$1.40 based the highest incremental cost for PCV delivery in a low-income country^30^ and a 25% increase and decrease in health service costs. Upper and lower bounds for alternative scenarios are included in tables 1 and 2.

We also conducted a probabilistic sensitivity analysis (PSA) with 1000 runs. Parameters included in the PSA included population, disease burden parameters for each outcome including incidence, cases, hospitalisations, deaths as relevant, illness duration, vaccine impact, treatment costs, incremental delivery costs and wastage. Online supplemental appendix table 1A in the appendix details the parameters, ranges, distributions and sources.

10.1136/bmjgh-2021-007211.supp1 Supplementary data

### Ethical considerations

The Gambia Government/Medical Research Council Joint Ethics Committee (GG/MRC JEC) approved the surveillance study (number 1087). Parents or guardians gave written informed consent for all surveillance procedures. The economic burden of disease study was approved by the GG/MRC JEC (number 1211) and the PATH Research Ethics Committee (HS 560). Parents or guardians gave written informed consent for questionnaires associated with costs of pneumococcal disease.

### Patient and public involvement

A public meeting was held in Basse to present the beginning of the surveillance study. In 2011, a series of four village meetings were held throughout the study area to facilitate discussion and awareness of the study. The study design and outcome measures did not involve public involvement. Dissemination of results involved information delivered by radio, five community meetings and a joint press conference with the Ministry of Health.

## Results

### Base case scenario

Compared with no vaccination, we estimated that the use of PCV in The Gambia between 2011 and 2030 would avert 117 000 total disease episodes including non-severe and severe pneumonia, pneumococcal sepsis and pneumococcal meningitis and its sequelae. We estimated that the PCV programme would avert 9000 outpatient pneumonia visits and 88 000 total hospitalisations due to pneumonia, pneumococcal sepsis and pneumococcal meningitis. The PCV programme is estimated to avert more than 3300 deaths from pneumonia, pneumococcal sepsis and pneumococcal meningitis over this period. Nearly 100 000 DALYs are expected to be averted.

Averting outpatient and inpatient medical visits is estimated to reduce the cost of medical care from the government perspective by nearly US$4 million over the period compared with no vaccination. If we include costs incurred by households, the total societal cost of care averted totals nearly US$7.3 million. Conversely, the additional costs of the vaccine programme are nearly US$2 million over the period. Approximately 40% of the costs of the vaccine programme are attributable to PCV and supplies, whereas 60% of the costs are associated with delivery. In the base case scenario, the government cost savings attributable to averted disease exceed vaccine programme costs by nearly US$2 million indicating that the PCV programme is cost saving in The Gambia. Table 3 contains details of health and economic outcomes.

**Table 3:** Health and economic outcomes among 20 cohorts vaccinated over 2011–2030 (2017 US$)

	No vaccine	With vaccine	Averted
**Total cases**	573 685	456 962	116 724
Total non-severe pneumonia cases	211 532	200 955	10 577
Total severe pneumonia cases	347 831	253 917	93 914
Total pneumococcal meningitis cases	1189	59	1129
Total pneumococcal meningitis sequelae	654	33	621
Total pneumococcal sepsis cases	12 480	1997	10 483
**Total outpatient visits**	159 303	150 479	8554
**Total hospitalisations**	297 927	210 138	87 789
Total pneumonia hospitalisations	285 222	208 212	77 010
Total pneumococcal meningitis hospitalisations	975	49	926
Total pneumococcal sepsis hospitalisations	11 730	1877	9854
**Total deaths**	13 633	9186	3336
Total pneumonia deaths	12 355	9019	3336
Total pneumococcal meningitis deaths	338	17	321
Total pneumococcal sepsis deaths	940	150	790
**DALYs (discounted**)	290 587	194 837	95 750
**Total government healthcare costs**			
(discounted)	**$13 696 698**	**$9 739 493**	**$3 957 205**
Total outpatient visit costs	$801 121	$761 065	$40 056
Total hospitalisation costs	$12 895 577	$8 978 428	$3 917 149
**Total societal healthcare costs**			
(discounted)	**$24 068 433**	**$16 804 536**	**$7 263 897**
Total outpatient visit costs	$2 234 169	$15 727 762	$661 407
Total hospitalisation costs	$21 834 264	$15 231 774	$6 602 490

DALY, disability-adjusted-life-year.

**Figure 1:**
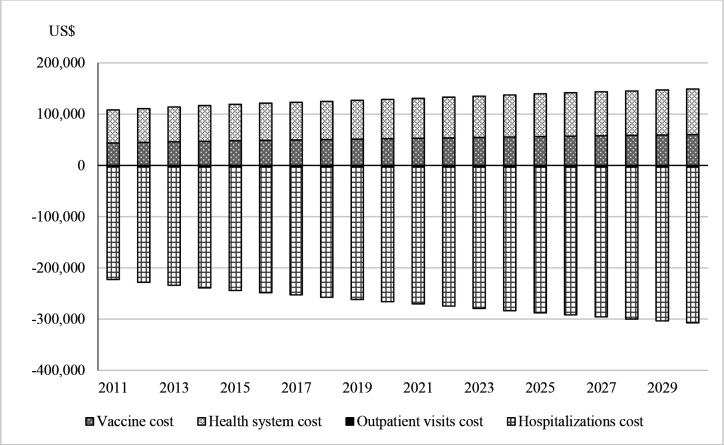
Budget implications (base case scenario from the government perspective, all figures undiscounted). Vaccine programme costs are positive and thus above the horizontal axis representing budget neutrality. Outpatient and hospitalisation costs averted are represented below the horizontal axis. Outpatient visit costs averted are not visible due to small magnitude.

In addition to the discounted outcomes discussed above, it is useful to examine undiscounted financial outcomes to inform budgets. Figure 1 displays the annual cost of the vaccine programme (positive values) and the costs of care averted (negative values) across the 20-year duration of the analysis. The undiscounted annual cost of the vaccine programme is approximately US$130 000 on average. Incorporating cost savings to the government from averted outpatient visits and hospitalisations, however, results in net savings of approximately US$140 000 per year on average.

### Alternative scenarios

**Figure 2:**
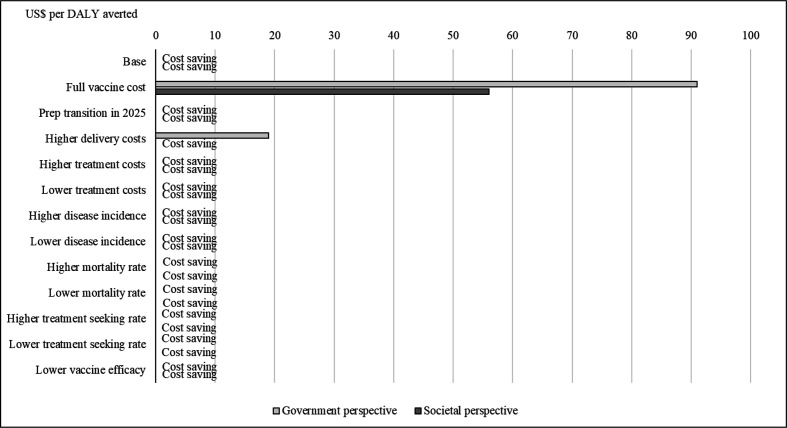
Scenario analysis results: cost per DALY averted in US$ (costs and benefits discounted). DALY, disability-adjusted-life-year.

Figure 2 illustrates how changes in key input parameters influence the ICER. Most of the scenarios we examined resulted in no change to the primary finding that the PCV programme in The Gambia was likely to be cost saving over a 20-year period. If The Gambia was to take full responsibility for the cost of PCV, however, the vaccination programme would no longer be cost saving. In this scenario, the cost/DALY averted would be US$91 from the government perspective and US$56 from the societal perspective. From the government perspective, the vaccine programme remains cost saving until the PCV copayment exceeds US$0.66 per dose. In another scenario with higher delivery costs, the vaccine programme would no longer be cost saving from the government perspective (US$19/DALY averted) but would remain cost saving from the societal perspective. The PCV programme is estimated to be cost saving in The Gambia in all other scenarios that examined earlier advancement to Gavi’s preparatory transition phase, higher or lower disease incidence, higher or lower case fatality rates, higher or lower treatment seeking rates, treatment costs or lower vaccine impact. online supplemental appendix figures 1A, 2A, and 3A in the appendix examine annual programme costs and savings for scenarios in which The Gambia pays the full vaccine price, transitions to preparatory transition in 2025 or incurs higher delivery costs.

The PSA results in findings similar to the alternative scenarios. From a government perspective, 891 of 1000 runs resulted in vaccination being cost saving in The Gambia. All runs found vaccination to be cost saving from the societal perspective. Scatter plots and a cost-effectiveness acceptability curve are in the appendix as online supplemental appendix figure 5A, 6A and 7A.

## Discussion

To the best of our knowledge, this is the first study to indicate that PCV immunisation, at actual levels of payment for vaccine, can be cost saving at the country level. We used local empiric data on the impact of the Gambian PCV programme, economic burden of disease and immunisation programme costs, and found that the introduction of PCV at the Gavi copayment cost of US$0.20 per dose would result in overall government cost savings of US$4 million over 20 years. This is some of the strongest evidence of the impact of PCV immunisation and demonstrates the success achieved by one country—giving reassurance to other low-income countries with per capita GNI in the lowest Gavi category.

This analysis demonstrates that the PCV programme is projected to avert a substantial health and economic burden of disease in The Gambia. Our finding, that under most scenarios, the PCV programme is likely to be cost saving, means that the country is projected to experience reduced disease burden (88 000 hospitalisations and 3300 deaths) and also save economic resources (US$4 million in costs of medical care). Our estimates suggest that the PCV programme (costing US$2 million) will remain cost saving for The Gambia until the country pays approximately US$0.66 per dose. The Gambia’s current GNI per capita is US$750 and, over the 20-year time horizon of our study, is highly unlikely to increase to become ≥US$1036 when the Gavi copayment would begin to increase. Even after The Gambia incurs the full cost of vaccine purchase when GNI per capita is >US$1580, however, the vaccine programme is likely to remain highly cost effective.

In terms of cost effectiveness, our analysis corroborates the existing literature that demonstrates PCV to be cost-effective in low-income and middle-income countries.^12 19^ Furthermore, the results suggest that PCV may be a better buy than indicated by previous economic studies undertaken in The Gambia and other low-income countries. For example, Kim *et al* report cost effectiveness ratios in the range of US$600 per DALY in The Gambia.^17^ There are, however, several differences in model inputs that vary between this and our analysis: vaccine price, vaccine impact, mortality burden and treatment costs. Our vaccine price reflects the price paid by the country rather than the full cost of the vaccine. In addition, PCV impact, mortality estimates and treatment cost data in our model are based on local empiric data; whereas, previous analyses relied more heavily on international estimates and the necessary assumptions. If we adjust our price, mortality and treatment cost inputs to align with Kim *et al* more closely, we estimate approximately US$300 to avert one DALY. While changing a few parameters brings us closer to the findings of Kim *et al*, we believe much of the remaining difference can be explained by modelling approach, the use of local vaccine impact data and numerous smaller differences in methods and data inputs. Other studies based on Kenya, Laos, Cambodia, Haiti and The Gambia suggest that the introduction of PCV would be cost-effective but not necessarily cost saving. Comparing our country-level cost saving finding with these other low-income countries suggests a qualitative difference in cost-effectiveness at different income levels or levels of cofinancing. Countries in the lowest Gavi category may be more likely have cost-saving PCV programmes compared with those with GNI per capita ≥US$1036, at which level the PCV copayment begins to increment each year, or when GNI per capita is >US$1580 and countries transition to the Gavi tail price for PCV. This difference is primarily related to the Gavi copayment per dose. A Ugandan study using assumed inputs did conclude that vaccination would be cost saving with an assumed vaccine price of US$0.15.^16^ Our finding using empiric data supports this and other earlier modelling studies based on assumed inputs in low-income countries that the threshold vaccine cost for PCV to be cost-saving at country-level is around US$0.5 to US$1 per dose. This evaluation is most sensitive to rates of child mortality.^31^ Even when the use of PCV is not cost-saving, all previous studies have found it highly cost effective in low-income countries.

In addition to comparisons with the existing literature, we systematically examined the inputs most likely to influence our results through scenario analysis. We examined change in disease burden, vaccine impact and changes in various cost inputs. While results from these scenarios vary in terms of the overall impact of the vaccine programme, costs and incremental cost effectiveness ratio (ICER), findings consistently show that PCV is impactful and highly likely to be cost saving in The Gambia. The base scenario results suggest PCV to be both less costly and more effective than no vaccination. Thus, PCV would be a high value investment under any cost effectiveness threshold. While ICERs are now less commonly compared with an international income per capita threshold even under the least favourable scenario in which The Gambia pays the full vaccine cost, the ICER (US$91/US$56 per DALY averted from the government/societal perspective) is still less than 15% of per capita GNI meaning it is highly cost effective by nearly all measures.

Many low-income and middle-income countries are facing increasing budget pressure, especially those that are transitioning from Gavi support. While The Gambia can still rely on support from Gavi for the foreseeable future, carefully selecting cost-effective and affordable interventions is essential. In a limited budget environment, PCV appears to be an impactful and cost-saving intervention. Our findings also align with a recent analysis from Kenya showing that continuing the PCV programme after transitioning from Gavi support, although not cost-saving, is highly cost-effective.^19^

This analysis has several limitations. It utilises a commonly used model that is static and does not account for herd protection, disease burden shifting to older age groups or serotype replacement.^32^ The vaccine impact inputs in the model, however, were designed to capture reductions in disease among unvaccinated children and captured herd effects by evaluating reductions in total disease burden, including unvaccinated children and those that received partial vaccination. We omitted disease burden and PCV impact in older children and adults due to associated herd protection as well as the potential for disease to shift to older age groups. This is a limitation of our approach. Findings from Kenya and The Gambia, although with limited precision, do suggest that PCV is associated with herd protection in older children and adults.^8–10^ Our approach does not allow us to assess serotype replacement over time. However, our modelling is informed by 10 years of population-based surveillance data which partially alleviates concerns of serotype replacement over this time. Overall, it is difficult to project how the use of a static model may affect our results. The omission of herd protection in older age groups will have led to some underestimation of the cost-effectiveness while our inability to fully capture serotype replacement or a potential age shift to older adults would have the opposite effect. While the authors recognise the limitations of a static model, we proceeded within a static framework due to benefits of easier interpretation and fewer input data requirements

We also exclude relative coverage from our analysis meaning that we assume independence between those that receive vaccination and those most likely to become ill. In both cases, we believe the impact on the analysis is small due to high vaccination coverage in The Gambia. Given that our local estimates of systems costs associated with vaccine delivery are low relative to the international literature, we also examine scenarios with greater programmatic costs. In addition, we extrapolate disease surveillance from one region to the entire country and recognise the limitations to this approach.

One of the key strengths of this work is the extensive engagement with the health system in The Gambia and the local context. This study benefits from a wealth of local data, including disease surveillance, health system utilisation, vaccine impact and programmatic and health system costs. Importantly, our findings are highly relevant to the 30 very low-income countries that are unlikely to begin the process of transition from Gavi support in the next 10 years. The budgetary challenges to make PCV copayments to Gavi are worthwhile as overall government spending is likely to be reduced by vaccination. Very low-income countries that have not yet introduced PCV due to problems of finance, vaccine coverage or other operational factors (eg, South Sudan, Somalia and Guinea) can be motivated to overcome these challenges given the likely cost savings if able to introduce PCV with reasonable coverage. In general, policymakers in low-income countries should have confidence in their decision to introduce PCV and maintain their vaccination programme in the coming years.

## Data Availability

All data relevant to the study are included in the article or uploaded as supplementary information.
