# Supplementary material for: Pneumococcal conjugate vaccination in The Gambia: health impact, cost effectiveness and budget implications
Source: BMJ Glob Health. 2021 Dec 16;6(12):e007211. doi: 10.1136/bmjgh-2021-007211 (PMC8679103; doi:10.1136/bmjgh-2021-007211)
Supplement: Supplementary data [file bmjgh-2021-007211supp001.pdf]

Appendix

Figure 1 from main text. Budget implications (Base case scenario from the government perspective, figures undiscounted)

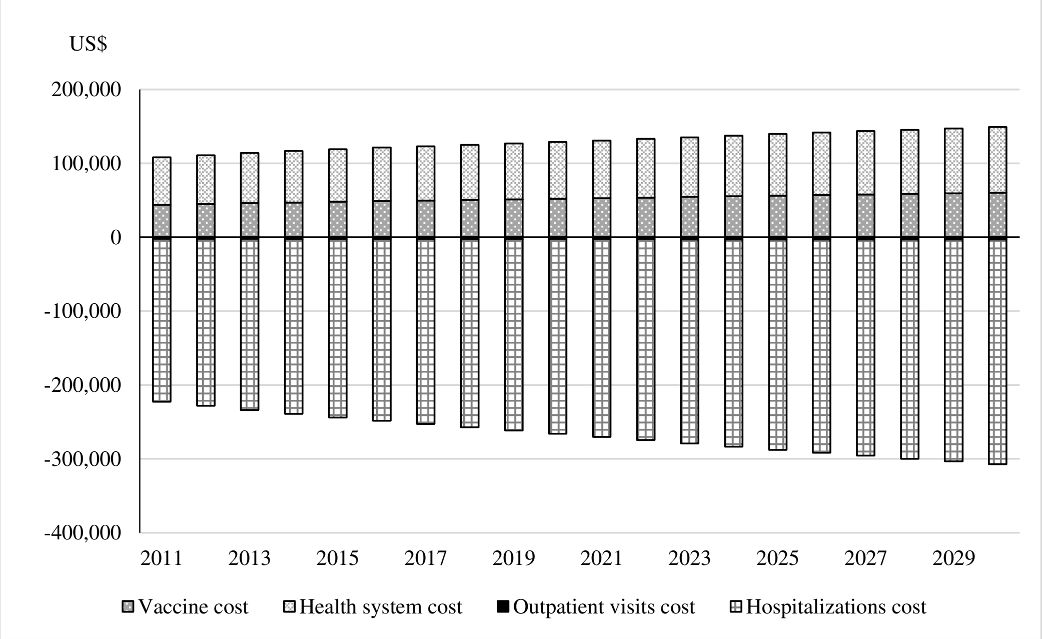

Figure 1A. Budget implications: Full vaccine cost scenario ($2.95 per dose) from the government perspective, all figures undiscounted

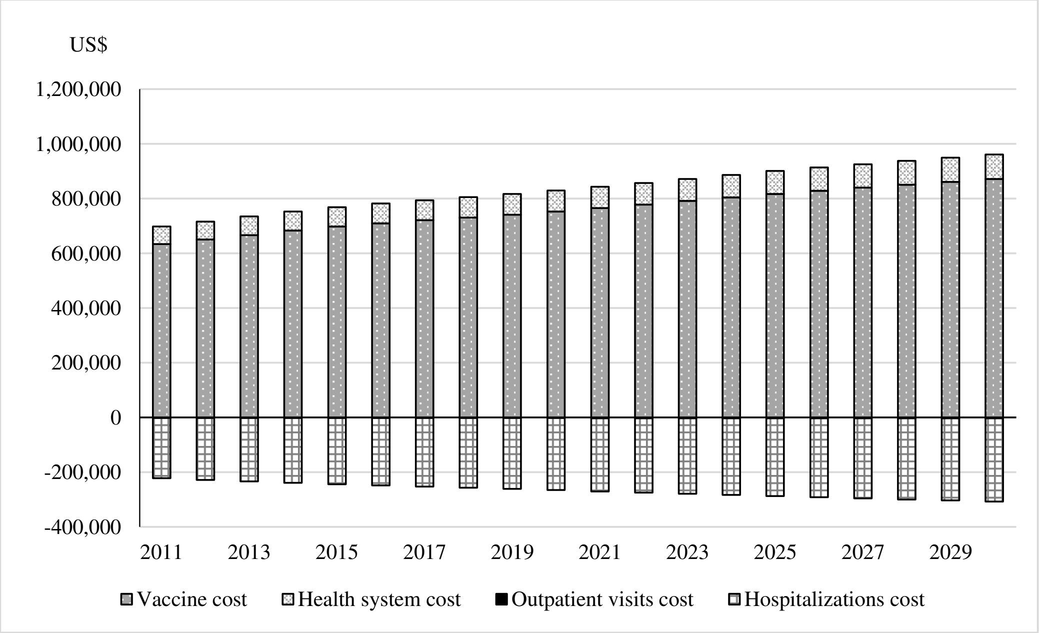

Figure 2A. Budget implications: Higher delivery cost scenario ($1.40 per dose) from the government perspective, all figures undiscounted

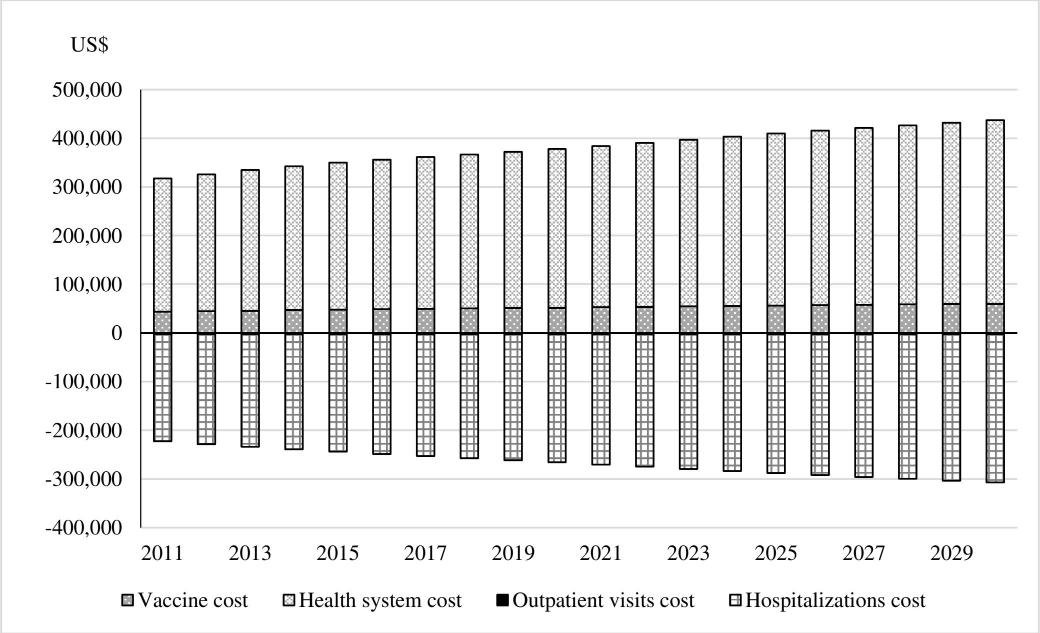

Figure 3A. Budget implications: The Gambia enters Gavi preparatory transition in 2025 scenario from the government perspective, all figures undiscounted

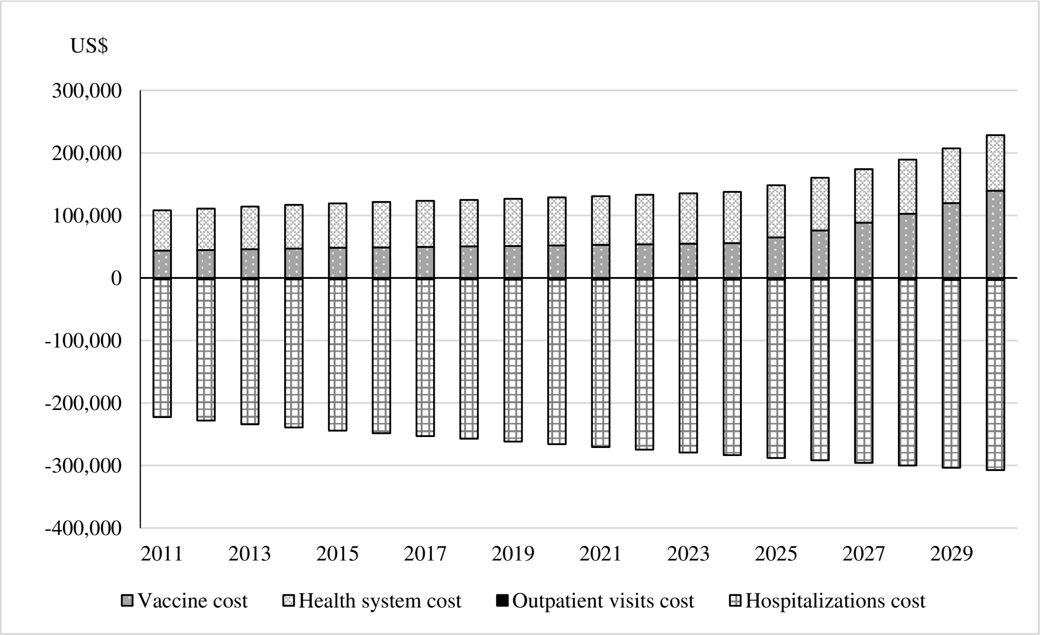

Pecenka C, *et al. BMJ Global Health* 2021; 6:e007211. doi: 10.1136/bmjgh-2021-007211

Figure 4A. Model schematic

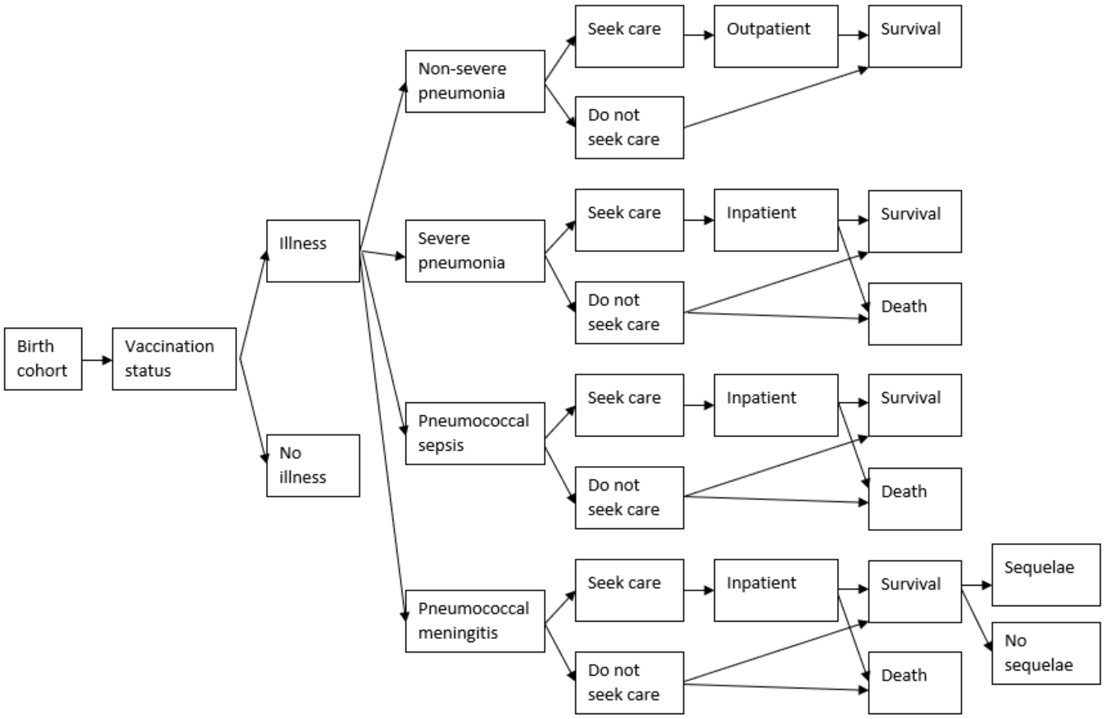

Non-severe and severe pneumonia are all cause disease categorizations while pneumococcal sepsis and pneumococcal meningitis are pathogen specific. These categorizations were selected to reflect the available evidence on impact and disease burden.

Note: Non-severe pneumonia was defined according to standardized surveillance criteria, was not cause-specific, and when patients were treated as outpatients. Severe pneumonia was defined according to standardized surveillance criteria, was not cause-specific, and when patients were treated in hospital. Pneumococcal sepsis was defined as a patient with *Streptococcus pneumoniae* isolated from the blood or lung. Pneumococcal meningitis was defined as a patient with *S. pneumoniae* isolated from cerebrospinal fluid.

Figure 5A. Cost-effectiveness of PCV in The Gambia from a government perspective

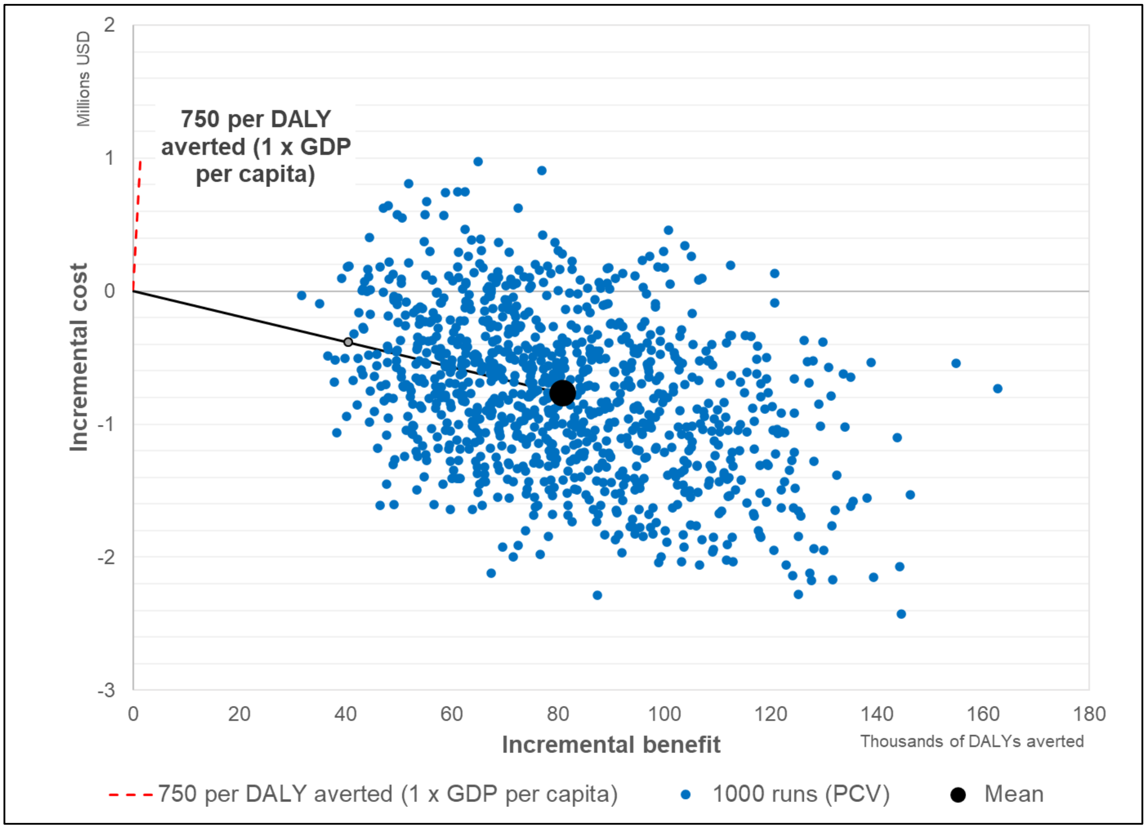

Scatterplot of 1000 model runs from the government perspective. Most of the plotted points are in the fourth quadrant indicating positive incremental benefit and negative incremental costs. Points in quadrant four are cost-saving.

Figure 6A. Cost-effectiveness of PCV in The Gambia from a societal perspective

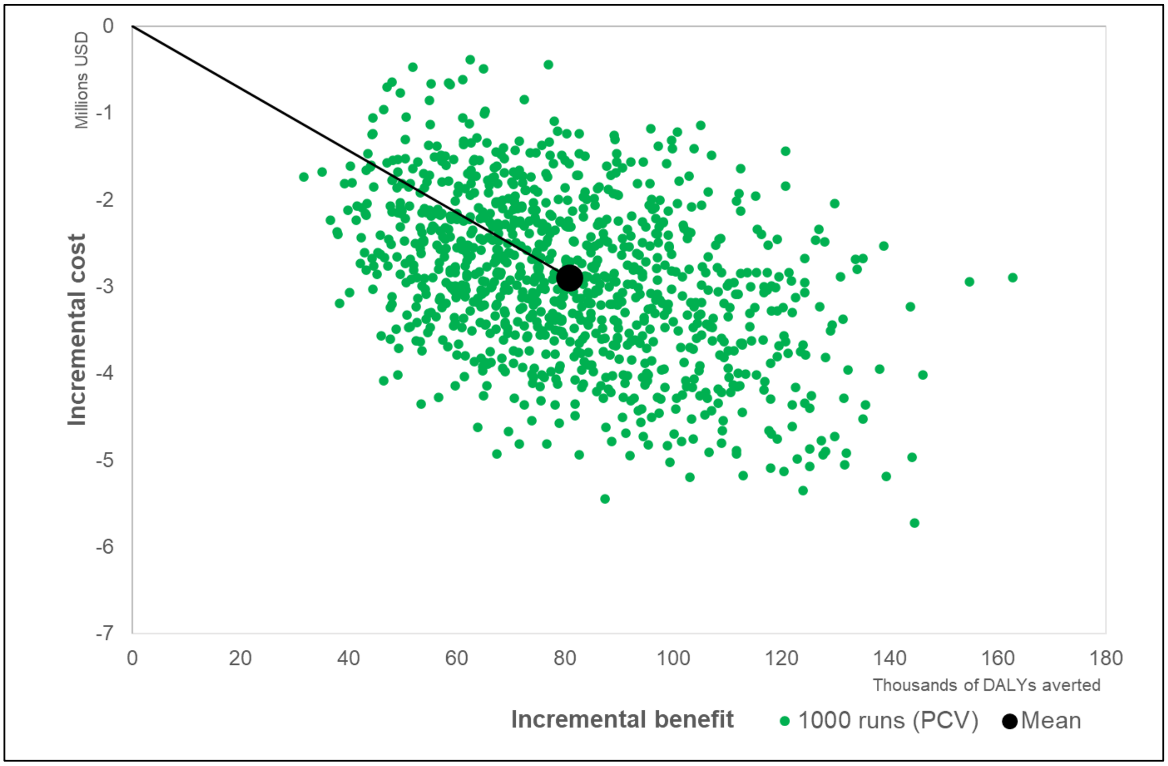

Scatterplot of 1000 model runs from the societal perspective. All of the plotted points are in the fourth quadrant indicating positive incremental benefit and negative incremental costs. Points in quadrant four are cost-saving.

Figure 7A. Probability that PCV is cost-effective at different willingness-to-pay thresholds, from a government perspective

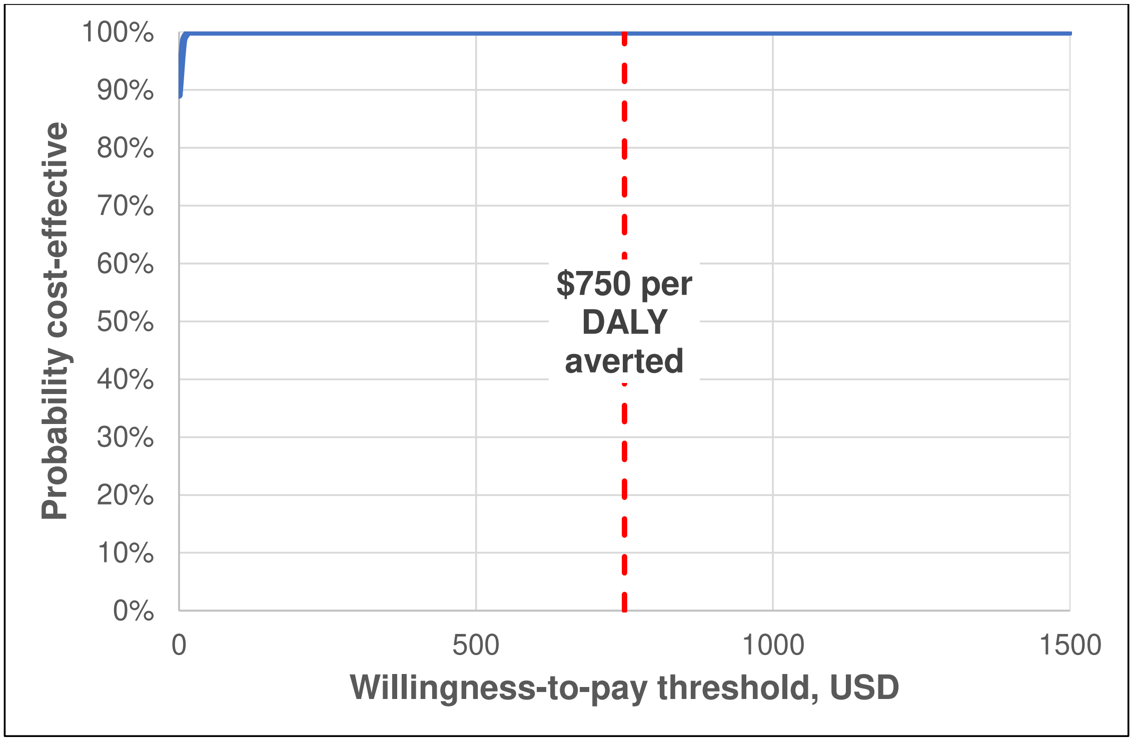

Cost effectiveness acceptability curve showing probability that intervention is cost-effective at different government willingness-to-pay thresholds. The cost per Disability Adjusted Life Year (DALYs) averted are often compared to per capita income. The current GNI per capita is $750 in The Gambia represented by the dashed line. The line near the top of the figure indicates that PCV is highly probable to be cost-effective at almost any willingness-to-pay threshold in The Gambia.

Pecenka C, *et al. BMJ Global Health* 2021; 6:e007211. doi: 10.1136/bmjgh-2021-007211

Table 1A. Parameters included in the probability sensitivity analysis, base, ranges and distributions

Parameter	Base estimate	Range		Source/s	Distribution	Notes
		Low	High			
**Target population**	Country specific	low variant	high variant	UNPOP. World Population Prospects - 2017 revision: https://population.un.org/wpp/ Accessed July 14, 2020.	Beta-Pert	
**Annual incidence per 100,000 age <5 years**						
Non-severe pneumonia cases	2,623.5	2,361.3	2,905.5	[8, 26]	Beta-Pert	
Non-severe pneumonia outpatient visits	1,967.6	1,705.0	2,230.0	[8]	Beta-Pert	
Severe pneumonia cases	4,313.9	3,992.1	4,655.5	[8, 26]	Beta-Pert	
Severe pneumonia hospitalisations	3,537.4	3,106.0	3,969.0	[8]	Beta-Pert	
Pneumonia deaths	168.7	83.58	337.32	[8]	Beta-Pert	
Pneumococcal meningitis cases	14.7	2.2	37.0	[8, 26]	Beta-Pert	
Pneumococcal meningitis hospitalisations	12.1	1.8	30.3	[8]	Beta-Pert	
Pneumococcal meningitis deaths	4.6	0.7	11.4	[8]	Beta-Pert	
Pneumococcal meningitis sequelae cases	8.1	1.2	20.3	[3]	Beta-Pert	
Pneumococcal sepsis cases	154.8	110.5	211.0	[8, 26]	Beta-Pert	
Pneumococcal sepsis hospitalisations	145.5	103.9	198.4	[8]	Beta-Pert	
Pneumococcal sepsis deaths	12.8	6.4	25.6	[8]	Beta-Pert	
**Mean duration of illness (in days)**						
Non-severe pneumonia	6	3	12	Assumption	Beta-Pert	0.5 and 2X base estimate

						for all durations
Severe pneumonia	9	4.5	18	Assumption	Beta-Pert	
Pneumococcal meningitis	13	6.5	26	Assumption	Beta-Pert	
Pneumococcal meningitis sequelae	10 years	5 years	20 years	Assumption	Beta-Pert	
Pneumococcal sepsis	10	5	20	Assumption	Beta-Pert	
**Impact on disease**						
Non-severe pneumonia	5%	3.75%	6.25%	[8] and assumption	Beta-Pert	+/- 25% up to 100% for all
Severe pneumonia	27%	20.25%	33.75%	[8] and assumption	Beta-Pert	
Pneumococcal meningitis	95%	71.25%	100%	[8] and assumption	Beta-Pert	
Pneumococcal sepsis	84%	63%	100%	[8] and assumption	Beta-Pert	
**Health service costs**						
Government cost per outpatient pneumonia visit	$6.82	$3.68	$7.45	[3]	Beta-Pert	
Societal cost per outpatient pneumonia visit	$13.82	$5.74	$16.42	[3]	Beta-Pert	
Government cost per pneumonia hospitalisation	$57.44	$34.29	$64.89	[3]	Beta-Pert	
Societal cost per pneumonia hospitalisation	$97.48	$56.55	$116.42	[3]	Beta-Pert	
Government cost per bacterial meningitis hospitalisation	$110.94	$67.05	$124.68	[3]	Beta-Pert	
Societal cost per bacterial meningitis hospitalisation	$152.86	$105.83	$209.32	[3]	Beta-Pert	

BMJ Publishing Group Limited (BMJ) disclaims all liability and responsibility arising from any reliance placed on this supplemental material which has been supplied by the author(s)

Government cost per pneumococcal sepsis hospitalisation	$77.73	$44.97	$106.64	[3]	Beta-Pert	
Societal cost per pneumococcal sepsis hospitalisation	$128.99	$66.70	$173.78	[3]	Beta-Pert	
Annual societal cost per bacterial meningitis sequelae	$44.88	$0	$44.88	[3]	Beta-Pert	
**Incremental cost per dose delivered**	$0.33	$0.17	$0.66	[3] and assumption	Beta-Pert	0.5 and 2X base estimate
**Vaccine wastage**	5%	2.5%	10%	[29] and assumption	Beta-Pert	0.5 and 2X base estimate

91
